# Riparian Collembola (Hexapoda) communities of northern Moldova, Eastern Europe

**DOI:** 10.3897/zookeys.724.12478

**Published:** 2017-12-21

**Authors:** Galina Buşmachiu, Ľubomír Kováč, Dana Miklisová, Wanda Maria Weiner

**Affiliations:** 1 Institute of Zoology, Academy of Sciences of Moldova, Academiei str. 1, 2028 Chişinău, Republic of Moldova; 2 Institute of Biology and Ecology, Faculty of Science, P. J. Šafárik University, Moyzesova 11, 040 01 Košice, Slovakia; 3 Institute of Parasitology SAS, Hlinkova 3, 040 01 Košice, Slovakia; 4 Institute of Systematics and Evolution of Animals, Polish Academy of Sciences, Sławkowska 17, Pl–31 - 016 Kraków, Poland

**Keywords:** Soil fauna, riverine ecosystems, species diversity, community ecology

## Abstract

Collembola were studied in a well-preserved riverine section of the Prut River in the Republic of Moldova. The study was focused on species diversity and habitat preferences of the particular species at two localities. Riparian habitats of the Prut River near Branişte included open river bank, forest belt and meadow, and the shore of Lake Costeşti-Stânca included meadow, pasture and shrub vegetation. In total 77 collembolan species were recorded, of which *Neanura moldavica* and *Arrhopalites prutensis* were endemic to Moldova. Comparative analyses showed a specific community composition at Branişte, with *Anurida ellipsoides* and *Mesaphorura macrochaeta* being abundant on the river bank and *Hemisotoma thermophila* in the meadow. In contrast, the forest plantation at the same locality was similar to the shrub-land in Costeşti, with the common species *Mesaphorura critica*, *M. yosii*, *Deutonura albella* and *Isotomiella minor*. Hygrophilous species preferred the habitats of the river section in Branişte, with quiet backwaters, to the artificial shoreline of the large lake. Species diversity was relatively high in the natural meadow and forest in Branişte and also in shrub-land on the lake shore. The present study documented relatively high collembolan species diversity at the shoreline and running water sections in the upper catchment area of the Prut River in Moldova that involve naturally valuable inundated habitats of Eastern Europe.

## Introduction

Floodplain forests and wetlands are highly dynamic ecosystems that are very dependent on the flows and sedimentation patterns of their adjacent rivers, acting as a link between land and water (Hughes 2003). Although many wetland organisms are widespread, some riverine systems are known for their high levels of endemism (Primack 2010).

Collembola make up one of the most significant and important groups that can be found in the soil and sandy sediments of riverbanks, wetlands and floodplain forests. Deharveng and Lek (1995) noted high Collembola diversity in riparian habitats of the Pyrenean massif. The Collembola communities of riparian habitats and wetlands are primarily affected by differences in the hydrologic regime and moisture gradient (Rusek 1984, Rusek et al. 2009, Sterzyńska et al. 2014), especially by the duration of inundation (Russell et al. 2004, Russell and Griegel 2006) and also by the vegetation structure (Sterzyńska 2009, Sterzyńska et al. 2014). After inundated soils are dried out, the overall Collembola diversity and abundance of hygrophilic and hygrotolerant species rapidly decline (Lessel et al. 2011), replaced by more xerotolerant and ubiquitous species (Marx 2008). Collembola predominate in later successional stages of wet meadows (Sterzyńska and Pilipiuk 1999). Generally, riparian communities of Collembola are very heterogeneous at small spatial scales, with different responses to flooding intensities.

Data in the literature on soil invertebrates of wetland and riparian ecosystems in Eastern Europe is still very limited. The present study was focused on Collembola communities of the Prut River in Moldova, a 953 km long river that rises in the Eastern Carpathian Mts in Ukraine at an altitude of 1600 meters. It is an important tributary of the Danube River and forms a border between Moldova and Romania that has been guarded and thus protected for more than 50 years. Preliminary studies of Collembola diversity in the riparian habitats of the lower section of the Prut River (Buşmachiu 2006, 2008) revealed 51 species concerning natural steppe, forest belts, grasslands and humid habitats on the shores of lakes Manta and Beleu. We assumed that the relatively well-protected floodplain forests and meadows in the upper Prut River catchment area could sustain valuable Collembola diversity.

The objective of this contribution was to reveal the species diversity and habitat preferences of individual species of Collembola occupying riverine habitats of the Prut River near Branişte and the shore of Lake Costeşti-Stânca, situated in northern Moldova. The study also aimed to provide a basic comparison of the communities of the individual riverine habitats. We expected the habitat type to have an important influence on the composition of Collembola communities in the natural riparian ecosystems.

## Materials and methods

### Study sites

**Figure 1.**
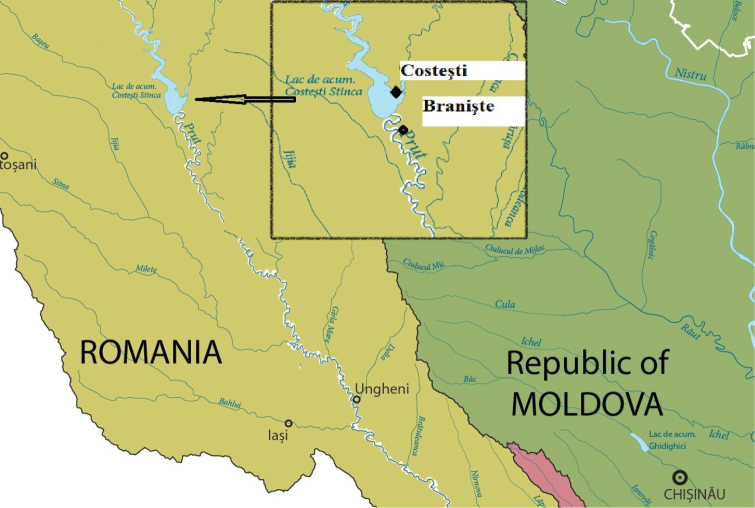
Location of the riparian habitats (colour: light green - upper catchment area of the Prut River, dark green - part of the Dniester (Nistru) River catchment area; the border between Romania and Moldova is represented by the Prut River).

**Figure 2.**
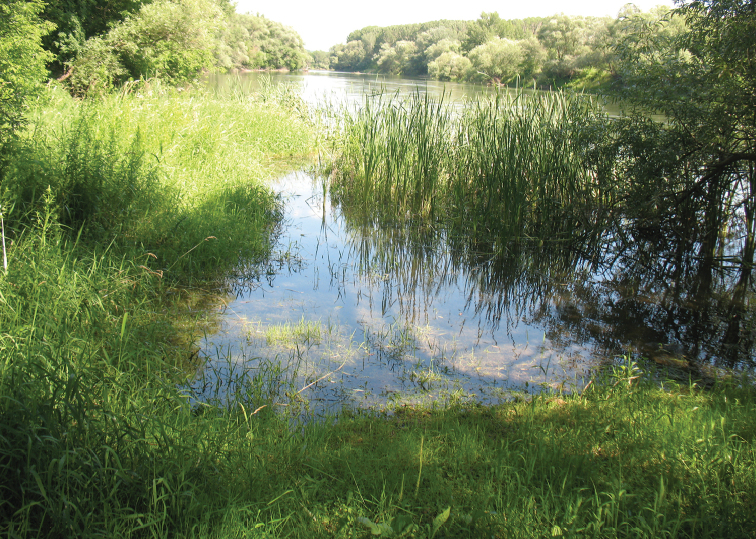
Bank of the Prut River near Branişte.

In 2013–2014 Collembola were studied in the riparian habitats of the Prut River upper catchment area, where fluvisols (FAO soil-type classification system) predominate with a clayey and silty clay structure and organic carbon content of 3.7–5.4%. The study included two localities with a distance of about 8 km between them (Fig. 1):

(1) Branişte village (47°49'1"N, 27°13'2"E; Fig. 2) - bank of the Prut River near the water; the level in July 2013 and June 2014 was high, sporadically flooding the bank covered by grassy vegetation. Further information on the water level of the river and temporal floods are provided by the State Hydrometeorological Service (http://www.meteo.md/mold).

The habitats selected on the distance gradient from the river were the following:

i) BB – 2–3 m wide, partially flooded river bank covered abundantly with herbaceous vegetation;

ii) BF – a 25–30 m wide protection forest belt plantation consisting mostly of *Quercus robur* mixed with *Populus alba*, *P. nigra*, *Salix alba*, *S. triandra* and *S. purpurea*, with moss and wooden debris; litter and humus layer ca. 3 cm thick;

iii) BM – a 45-meter wide belt of meadows along the banks with embankments for flood protection that separate the river from the meadows and nearby lake.

**Figure 3.**
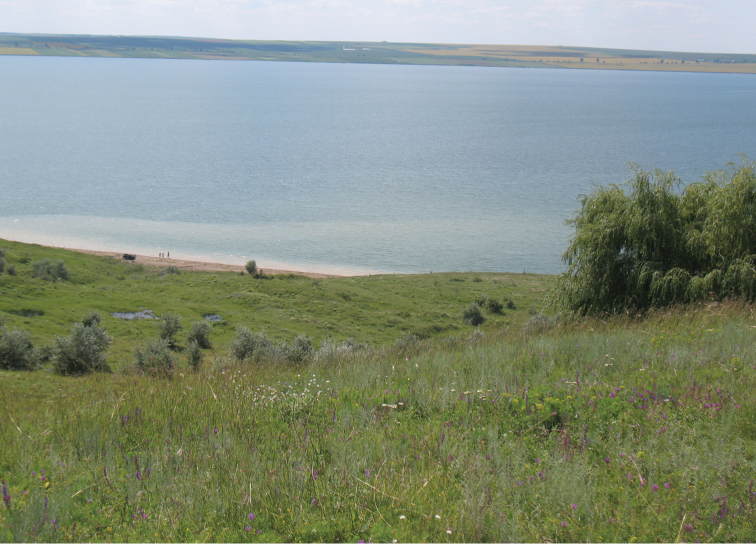
Shore of the Lake Costeşti-Stânca.

(2) Lake Costeşti–Stânca (47°55'1"N 27°08'3"E; Fig. 3), a water reservoir 70 km long and 59 km^2^ in area, situated on the Prut River between Romania (Stânca village) and the Republic of Moldova (Costeşti village). The lake shore consists of a dam built in 2007 with limestone rocks poured into the water about 5 m off the bank to protect the shoreline. The habitats on the distance gradient from the lake were the following:

iv) CSB – a 1–2 m wide bank of the lake partially covered by hygrophilous grassy vegetation, in some places covered by algae and decaying residues of *Phragmites australis* (Cav.), partially inundated in June 2014;

v) CSP – a narrow belt of pasture about 20–25 m distance from the bank, covered with herbaceous plants (*Urtica dioica* L., *Artemisia* sp., *Plantago* sp., etc.) and with occasional small shrubs (*Rosa canina* L.), on a steep bank > 3 m above the water level.

vi) CSUB – a belt of shrub-land about 20–35 m distance from the shore formed by groups of individual shrubs, covered with small shrubs (*Rosa canina* L., *Lycium barbarum* L.) with 1.80 m high thickets of *Onopordum acanthium* L. behind the pasture; litter and humus layer ca. 1.5 cm thick.

Previously, the study area was part of a buffer zone of the former USSR state border, which was fenced off and strictly limited to visitors. The riverine zone of the Prut River thus remained virtually untouched by humans after 1945; in 2010 the wire fence was removed.

### Sampling and extraction

The overall number of soil samples taken at Branişte and Costeşti was 25 and 27, respectively. The samples consisted of soil cores of size 5 × 5 × 5 cm taken by the first author in July and November 2013, and in June and July 2014; the sampling design is specified in Table 1. Some epiedaphic collembolan specimens were collected in the Branişte meadow and Costeşti shrub-land using an exhauster to catch epigeic species that are underestimated by the soil sampling.

**Table 1.** Sampling design and Collembola community parameters in riparian habitats at Branişte and Costeşti–Stânca (Prut River) in 2013–2014.

Locality	Branişte										
Habitat	Bank		Forest		Meadow						
Month	Jul	Nov	Jul	Nov	Jun	Jul					
Year	2013	2014	2013	2013	2014	2014					
Sample nr.	6	2	6	3	3	5					
Nr. of individuals	219	47	347	256	43	67					
Species richness	4–8	6	11–18	10–13	15	12					

Locality	Costeşti – Stânca										
Habitat	Bank			Pasture				Shrub-land			
Month	Jul	Jun	Jul	Jul	Nov	Jun	Jul	Jul	Nov	Jun	Jul
Year	2013	2014	2014	2013	2013	2014	2014	2013	2013	2014	2014
Sample nr.	4	2	2	4	4	2	1	4	1	1	2
Nr. of individuals	294	101	94	166	360	259	29	195	113	121	184
Species richness	12–13	11	7	11–12	5–6	14–15	10	12–15	11	16	14–16

The collembolans were extracted from the soil using the flotation method according to Buşmachiu et al. (2015). The specimens were fixed in 96% ethyl alcohol, sorted in a binocular stereomicroscope, cleared in lactic acid and KOH and mounted on permanent slides using Marc André II solution. The specimens were identified to the species level using a LEICA 2500 phase contrast microscope and the following principally taxonomical sources: Bretfeld (1999), Potapov (2001), Thibaud et al. (2004), Fjellberg (1998, 2007), Dunger and Schlitt (2011) and Jordana (2012). Geographic distribution and ecological data of the species were excerpted from the same literature. Collembola life forms were analyzed according to Gisin (1943) and Rusek (2007); the habitat preferences of species are provided in Table 2.

**Table 2.** List of Collembola from the riparian habitats of the Prut River with overall numbers of individuals collected, biogeographic distribution (BD), life forms and ecological traits. Abbreviations: for locality and habitat see "Material and methods"; C – cosmopolitan, E – European, H – Holarctic, P – Palaearctic, M – Mediterranean, R – known from type locality only; e – epiedaphic, h – hemiedaphic, eu – euedaphic; * – species new to Moldova.

Species	Locality/Habitat						BD	Ecological traits	
	BB	BF	BM	CSB	CSP	CSUB		Life form	Habitat
**Poduridae**									
*Podura aquatica* Linné, 1758	3						C	e	hydrophile
**Hypogastruridae**									
*Ceratophysella engadinensis* (Gisin, 1949)		14			3	8	C	h	woodland
*Ceratophysella stercoraria (Stach, 1963)					2		H	h	woodland
*Ceratophysella succinea* (Gisin, 1949)		2		14			C	h	grassland
*Schoettella ununguiculata* (Tullberg, 1869)	1	65	7	2	1	2	H	h	eurytopic
*Willemia scandinavica* Stach, 1949		2					H	eu	interstitial
**Neanuridae**									
*Anurida ellipsoides* Stach, 1920	126	34					P	h	humid soils
*Anurida tullbergi* Schött, 1891	1						H	h	humid soils
*Deutonura albella* (Stach, 1920)		3			1	21	E	h	woodland
*Friesea truncata* Cassagnau, 1958				15	6	13	P	h	woodland
*Pseudachorutes sp.						1		h	
*Pseudachorutes subcrassus* Tullberg, 1871						1	P	h	woodland
*Micranurida pygmaea* Börner, 1901		3					C	h	woodland
*Neanura moldavica* Buşmachiu & Deharveng, 2008		4					R	e	woodland
*Neanura muscorum* (Templeton, 1835)		1					C	e	woodland
**Onychiuridae**									
*Protaphorura armata* (Tullberg, 1869)		5					C	h	eurytopic
*Protaphorura sakatoi* (Yosii, 1966)	9	167	8	144	512	58	E	h	eurytopic
**Tullbergiidae**									
*Doutnacia xerophila* Rusek, 1974		2					E	eu	interstitial
*Mesaphorura critica* Ellis, 1976	9	47	18	5	10	33	P	eu	grassland
*Mesaphorura florae* Simón et al., 1994	14	41			6	69	E	eu	interstitial
*Mesaphorura simoni Jordana, Arbea, 1994				1	1		E	eu	humid soils
*Mesaphorura hylophila* Rusek, 1982		18					P	eu	interstitial
*Mesaphorura macrochaeta* Rusek, 1976	38	4					C	eu	eurytopic
*Mesaphorura rudolfi Rusek, 1987					1		E	eu	grassland
*Mesaphorura sylvatica* Rusek, 1971	8	6				1	P	eu	woodland
*Mesaphorura yosii* (Rusek, 1967)	5	29		74	4	40	C	eu	grassland
*Metaphorura affinis* (Börner, 1902)	1		2	27	3	14	P	eu	grassland
*Stenaphorura metaparisi* (Traser & Weiner, 1999)				1		2	E	eu	grassland
**Isotomidae**									
*Folsomia manolachei* Bagnall, 1939		5	9				P	h	eurytopic
*Folsomides sp.					1			eu	
*Hemisotoma thermophila* (Axelson, 1900)			24	95	11		C	h	eurytopic
*Isotoma viridis* (Bourlet, 1839)			16	13	15	2	H	e	grassland
*Isotomiella minor* (Schäffer, 1896)	16	72	3	2	5	85	H	eu	woodland
*Isotomodes productus* (Axelson, 1906)			8	9	44	9	C	eu	grassland
*Isotomurus antennalis Bagnall, 1940					1		E	e	grassland
*Isotomurus* sp. juv.	31	1		3			-	e	woodland
*Parisotoma notabilis* (Schäffer, 1896)		39		1	69	116	C	h	eturytopic
*Proisotoma minuta* (Tullberg, 1871)						9	C	h	woodland
**Entomobryidae**									
*Entomobrya nigrocincta* Denis, 1923						4	E	e	woodland
*Entomobrya handschini* Stach, 1922					2		E	e	grassland
*Entomobrya quinquelineata* Börner, 1901			2				E	e	grassland
*Entomobrya marginata* (Tullberg, 1871)			1			4	E	e	eurytopic
*Entomobrya multifasciata* (Tullberg, 1871)						2	H	e	grassland
*Entomobrya violaceolineata* Stach, 1913			6				E	e	grassland
*Entomobrya* juv.		3			4	12			
*Heteromurus major* (Moniez, 1889)			9			11	M	h	woodland
*Heteromurus nitidus* (Templeton, 1835)					1		C	eu	eurytopic
*Orchesella albofasciata* Stach, 1960			18	3			E	e	humid soils
*Orchesella cincta* (Linnaeus, 1758)		1					H	e	woodland
*Orchesella multifasciata* Stscherbakow, 1898				1	6	4	E	e	eurytopic
*Orchesella orientalis* Stach, 1960			1				E	e	grassland
*Orchesella villosa (Geoffroy, 1762)				6			H	e	grassland
*Lepidocyrtus arrabonicus Traser, 2000				32			E	e	grassland
*Lepidocyrtus lignorum* (Fabricius, 1793)			2				H	e	woodland
*Lepidocyrtus paradoxus* Uzel, 1890			1				H	e	humid soils
*Lepidocyrtus violaceus* (Lubbock, 1873)			6		3		H	e	woodland
*Pseudosinella horaki* Rusek, 1985	1	13					E	h	woodland
*Pseudosinella imparipunctata* Gisin, 1953			1	23	61	54	E	h	grassland
*Pseudosinella octopunctata* Börner, 1901			5	7	12	2	C	h	grassland
**Tomoceridae**									
*Pogonognathellus flavescens* (Tullberg, 1871)		1					H	e	woodland
*Tomocerus vulgaris* (Tullberg, 1871)		1					C	h	woodland
**Cyphoderidae**									
*Cyphoderus albinus* (Nicolet, 1842)					2		P	eu	grassland
*Cyphoderus bidenticulatus* (Parona, 1888)				1	1	3	M	eu	grassland
*Oncopoduridae									
*Oncopodura crassicornis Shoebotham, 1911					8	4	P	eu	grassland
**Neelidae**									
*Neelus murinus* Folsom, 1896		15		2	3	4	C	eu	woodland
*Megalothorax minimus* Willem, 1900		1				1	C	eu	woodland
**Sminthurididae**									
*Sphaeridia pumilis* (Krausbauer, 1898)	1		15	7	10	11	C	h	eurytopic
*Stenacidia violacea (Reuter, 1881)			3				C	e	humid soils
**Arrhopalitidae**									
*Arrhopalites prutensis* Vargovitsh & Buşmachiu, 2015	4						R	eu	woodland
*Arrhopalites ulehlovae* Rusek, 1970	1						E	eu	woodland
*Pygmarrhopalites terricola* (Gisin,1958)		7					E	eu	woodland
**Katiannidae**									
*Sminthurinus aureus* (Lubbock, 1862)					1	2	P	e	grassland
*Sminthurinus bimaculatus* Axelson, 1902	1	2					P	e	humid soils
*Sminthurinus elegans* (Fitch, 1863)			1			4	E	e	grassland
**Bourletiellidae**									
*Deuterosminthurus* sp.			1	1		2	-	e	grassland
**Sminthuridae**									
*Sminthurus nigromaculatus (Tullberg, 1871)					4		H	e	grassland
*Sminthurus viridis* (Linné, 1758)		1	1			4	C	e	grassland
*Caprainea marginata* (Schött, 1893)						1	P	e	woodland
	**270**	**609**	**168**	**489**	**814**	**613**			
**Life forms** - dominance [%]									
epiedaphic	13.0	1.5	35.1	12.1	4.5	6.7			
hemiedaphic	51.4	57.6	46.4	62.9	84.4	50.0			
euedaphic	35.6	40.9	18.5	25.0	11.1	43.3			

### Data analyses

For the basic comparison of Collembola community structure between sites and habitats, data from the summer (June and July) of both years were used. Box-plots for number of specimens and species richness were depicted using STATISTICA for Windows version 12.0 (StatSoft, Inc. 2013). Non-metric multidimensional scaling (NMS) ordination processed average quantitative data to examine the community structure between 29 plots differentiated by locality (12 from B - Branişte and 17 from CS - Costeşti-Stânca) and habitat. The ordination covered 16 species in total; species with dominance < 1% were excluded from the analysis due to an unclear relationship with the studied habitats. The epiedaphic collembolan specimens collected by the exhauster in meadow and shrubland were also excluded from the analysis. Autopilot, with slow and thorough mode, and Sørensen (Bray-Curtis) distance, recommended for community data, were selected. After 250 randomized runs, a two-dimensional solution was accepted as optimal. PC-ORD software (McCune and Mefford 2011) was used for the NMS analysis.

## Results

### Collembola diversity

Altogether, 2963 individuals of Collembola belonging to 77 species were found in the habitats along the Prut River (Table 2): 1916 individuals were collected in the riverine habitats of Costești and 1047 individuals in that of Branişte. In spite of the difference between the numbers of individuals, the number of species was similar, i.e. 52 in Costeşti and 54 in Branişte. Although the sites are rather close to one another, being located on the same bank of the river, only 28 collembolan species were common to both.

According to the life forms, 26 collembolan species were epiedaphic, 22 hemiedaphic and 25 euedaphic. The largest portions were woodland (26) and grassland (25) species, followed by eurytopic species (10) and species preferring interstitial habitats (4). Moreover, the riparian communities of the Prut River area included seven hygrophilous and one hydrophilous (*Podura aquatica*) species, representing 10.3% of the total species number.

The majority of species have a wide geographic occurrence, namely European (29.5%), cosmopolitan (25.9%) and Palaearctic or Holarctic species (16.9%); two species have a Mediterranean distribution range and two species have a range limited to Moldova (*Neanura moldavica*, *Arrhopalites prutensis*).

Nine species, namely *Ceratophysella stercoraria*, *Mesaphorura rudolfi*, *M. simoni*, *Isotomurus antennalis*, *Orchesella villosa*, *Lepidocyrtus arrabonicus*, *Oncopodura crassicornis*, *Stenacidia violacea* and *Sminthurus nigromaculatus* were new for the fauna of the Republic of Moldova. Two species, *Folsomides* sp. and *Pseudachorutes* sp., are potentially new for science.

### Comparison of communities at localities and habitats

Collembola in Branişte were more abundant in the forest belt, and in Costeşti higher numbers of them were recorded in pasture and under shrubs. *Protaphorura sakatoi*, *Isotomiella minor* and *Mesaphorura critica* were abundant and frequent in all habitats of both localities, representing hemiedaphic and/or euedaphic life forms. Similarly, *Schoettella ununguiculata*, *Mesaphorura yosii*, *M. florae*, *Parisotoma notabilis*, *Neelus murinus* and *Sphaeridia pumilis* were present in all or most habitats, but in lower number of specimens. *Anurida ellipsoides* was dominant in Branişte, and together with *A. tullbergi*, *Micranurida pygmaea*, *Neanura moldavica*, *N. muscorum*, *Tomocerus minor*, *Pogonognathellus flavescens* and *Podura aquatica* occurred exclusively in this locality (Table 2). In contrast, *Oncopodura crassicornis*, *Cyphoderus albinus* and *C. bidenticulatus* were found only in pasture soil in Costeşti. *Friesea truncata*, *Pseudosinella imparipunctata* and *Isotomodes productus* were abundant and frequent in the riparian habitats near the lake.

The low bank of the Prut River covered by forest belt in Braniște was associated with a higher number of hygrophilous species (7) than the pasture and shoreline of the lake (2 species; Table 2); hygrophilous *Anurida ellipsoides* was abundant on the flooded river bank.

High dominance of a few species was observed in the pasture, specifically *Protaphorura sakatoi*, *Parisotoma notabilis* and *Pseudosinella imparipunctata*, which shared 54.1% of the total community dominance.

A high proportion of epiedaphic collembolans was observed in the meadow near the river, but this was affected by additional collection with an exhauster. These forms were relatively numerous on both banks of the river and lake, consisting mainly of isotomid and entomobryid collembolans. The pasture near the lake showed a very high proportion of hemiedaphic forms, dominated by *P. sakatoi*, *P. notabilis* and *P. imparipunctata*. A lower dominance of euedaphic forms was evident in open habitats, such as meadow and pasture, compared to other habitats.

**Figure 4.**
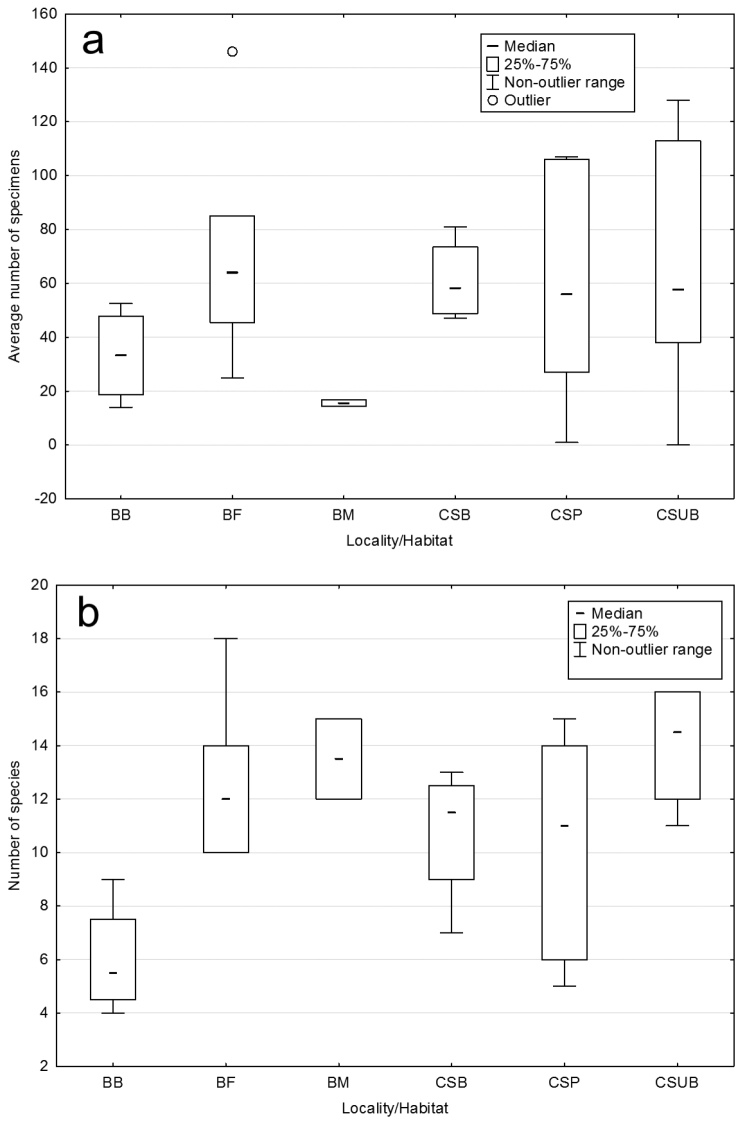
Box-plot diagram of Collembola specimens’ number (**a**) and species richness (**b**) in riparian habitats of the Prut River. For abbreviations see Materials and methods.

A box-plot diagram of Collembola number of specimens (Fig. 4a) showed higher median values in the forest belt in Branişte and in all three habitats near the lake, and lower values on the river bank and in the meadow near the river. The pasture and shrub-land near the lake had apparently higher non-outlier ranges of number of specimens compared with the others. A box-plot of species richness (Fig. 4b) documented the high median values in the shrub-land and meadow, while the lowest median was observed on the river bank. The pasture had a high non-outlier range of this community parameter.

**Figure 5.**
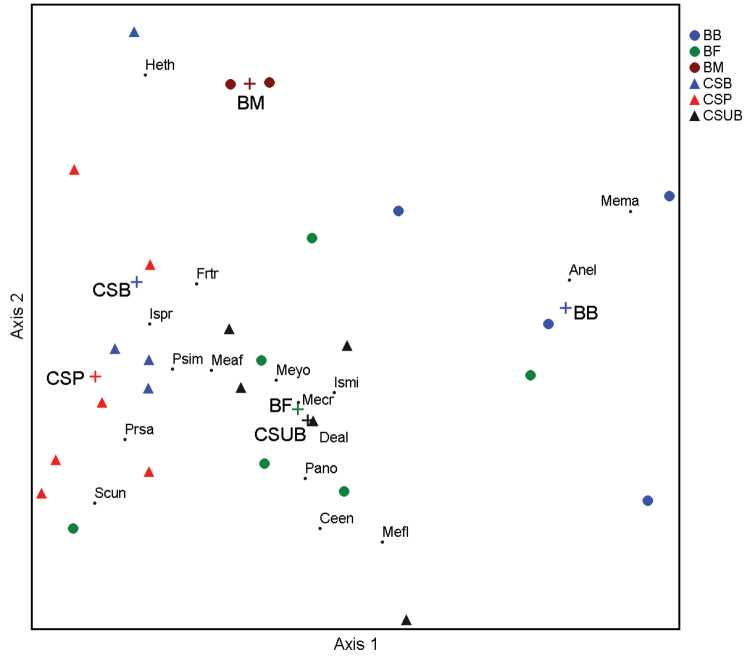
NMS ordination of Collembola species in riparian habitats of the Prut River from June–July 2013 and 2014, species with dominance ≥ 1% included. Colour circles and triangles represent localities and habitats, for abbreviations see Materials and methods, black dots represent species, for species abbreviations see Table 2. Variance explained by the first two axes was 38% and 34%, respectively.

An NMS ordination diagram (Fig. 5) outlines the Collembola community composition at localities/habitats during the summer (June and July) of both years. For the analysis, a two-dimensional solution was recommended by Autopilot and confirmed by the Monte Carlo permutation test, with a significant P < 0.005, a mean stress of 23.3 for real data and 250 runs for both real and randomized data. This best two-dimensional solution had a final stress of 17.8, P < 0.00001 after 110 iterations. The diagram shows that habitats along the river (Branişte) were rather dissimilar, with river bank and meadow communities showing a specific composition, where *Anurida ellipsoides* and *Mesaphorura macrochaeta* were abundant on the bank and *Hemisotoma thermophila* in the meadow. In contrast, forest plantation of the same locality was similar to the habitats of Costeşti, especially to the shrub-land with *Mesaphorura critica*, *M. yosii*, *Deutonura albella* and *Isotomiella minor* as the common species. In general, habitats near Lake Costeşti-Stânca had similar species composition.

## Discussion

The present study has some limitations that can be inferred from the non-uniform sampling design across the studied habitats. Nevertheless, several peculiarities of collembolan species diversity and distribution were observed at the natural inundation habitats of the Prut River and the shoreline of a nearby lake. The study revealed a relatively high overall species number of Collembola, which is likely the result of the long-term preservation of these riparian habitats over more than six decades. The forest belt and meadow on the river bank and the shrub-land near the lake were the most diverse in species.

According to Čarnogurský (1998) species of alluvial habitats have a wide geographic distribution (cosmopolitan, Holarctic and Palaearctic), while endemic species are missing in these habitats. Similarly, Sterzyńska (2009) found that in floodplains widely distributed species were the most numerous in all habitat types, which was also observed in the present study. In contrast, Deharveng and Lek (1995) revealed high Collembola diversity with a larger number of rare and/or endemic species in habitats along running waters, the pattern probably characteristic for higher mountains in southern Europe with high endemism in the local fauna. Regarding endemic species recorded during the present study, *Neanura moldavica* is a species widespread in Moldova, while *Arrhopalites prutensis* is recently known only from the natural floodplain forests of the Prut River. *Lathriopyga nistru*, *Micraphorura gamae* and *A. prutensis* inhabit the banks of the Prut and Dniester Rivers in Moldova (Buşmachiu and Deharveng 2008, Buşmachiu and Weiner 2013, Vargovitsh and Buşmachiu 2015) and probably represent local endemic species.

A portion of the hygrophilous species preferred habitats of the river section in Branişte with quiet backwaters to the shoreline of the large Lake Costeşti-Stânca. The studies carried out in riparian habitats of the Dniester River in Moldova (Buşmachiu and Weiner 2013), and in the similar habitats in Ukraine (Sterzyńska et al. 2014) showed the portion of hygrophilous species to be 13% and 12%, respectively. In the Arize massif (Pyrenees, France) Deharveng and Lek (1995) recognized 16% hygrophilous species that had high abundance. In contrast, hygrophilous species were not abundant in the Prut area, with the distinction probably associated with the different types of riverine habitats involved in both studies and the local climatic conditions (mountain and lowland climate, respectively).

At both localities three habitats were selected for the study, representing gradients in terms of their distance from the water. Herbaceous marginal vegetation near the water, forest belt (plantation) and meadow were in gradient from the river, and grassy shoreline vegetation, pasture and shrub-land in gradient from the lake. NMS ordination showed that natural riverine habitats (river bank, natural meadow) had specific communities, while the secondary habitats near the lake were similar in the composition with the forest plantation on the river bank. The difference between the natural and secondary riparian habitats was also striking in the low number of the common species. Moreover, the community structure in pasture, with the dominance concentrated in a few eurytopic or grassland species, indicated a disturbed habitat.

A box-plot diagram documented the tendency of riverine habitats with shrub and tree vegetation with a litter layer to have higher number of specimens and species richness compared to the others. The accumulation of organic residues and decaying wood also supported a greater variety of collembolan species. Moreover, ordination showed that both habitats had a similar community structure. On the other hand, the grassy bank of the river had apparently lower number of specimens and species richness and was a relatively extreme habitat occupied by a limited number of species adapted to inundations.

The present study implies that intact patches with natural, undisturbed floodplain forests and mostly intact riverine meadows and grasslands in the higher Prut River catchment area are very valuable natural habitats in terms of preservation of soil-fauna diversity in inundated habitats and wetlands of Eastern Europe.
